# Hydroxychloroquine dose-dependently reduces the risk of incident diabetes in primary Sjögren syndrome patients on glucocorticoids: a nationwide population-based cohort study

**DOI:** 10.1186/s13075-025-03542-7

**Published:** 2025-04-16

**Authors:** Wei-Sheng Chen, Hui-Ching Hsu, Tzu-Min Lin, Yu-Sheng Chang, Yi-Chun Lin, Tzu-Tung Kuo, Yu-Chuan Shen, Shu-Chuan Chen, Jin-Hua Chen, Hsiang-Yen Lee, Chi-Ching Chang

**Affiliations:** 1 Division of Allergy, Immunology, and Rheumatology, Department of Internal Medicine, Taipei Veterans General Hospital, National Yang Ming Chiao Tung University, Taipei, Taiwan; 2 Division of Allergy, Immunology and Rheumatology, Department of Internal Medicine, School of Medicine, College of Medicine, Taipei Medical University, 252 Wu-Hsing Street, Taipei, Taiwan; 3 Division of Rheumatology, Immunology, and Allergy, Department of Internal Medicine, Taipei Medical University Hospital, Taipei, Taiwan; 4 Division of Allergy, Immunology and Rheumatology, Department of Internal Medicine, Shuang Ho Hospital, Taipei Medical University, Taipei, Taiwan; 5 Division of Allergy, Immunology and Rheumatology, Department of Internal Medicine, Wang Fang Hospital, Taipei Medical University, Taipei, Taiwan; 6 Biostatistics Center, College of Management, Taipei Medical University, Taipei, Taiwan; 7 Department of Mathematics and Statistics, Idaho State University, Pocatello, ID USA; 8 Graduate Institute of Data Science, College of Management, Taipei Medical University, Taipei, Taiwan; 9 School of Public Health, Taipei Medical University, Taipei, Taiwan

**Keywords:** Diabetes, Hydroxycholoroquine, Sjögren Syndrome

## Abstract

**Background:**

Hydroxychloroquine (HCQ) is commonly used to treat Sjögren syndrome (SS). Glucocorticoids, which are commonly applied for managing primary SS (pSS), can disrupt glucose metabolism and increase diabetes mellitus (DM) risk. HCQ reduces DM risk in systemic lupus erythematosus and rheumatoid arthritis.

**Objective:**

This study aimed to investigate the relationship between HCQ and glucocorticoids in the incidence of new-onset diabetes in pSS.

**Methods:**

This nationwide population-based cohort study identified patients diagnosed with pSS from the Taiwan’s National Health Insurance Research Database from 2006 to 2015. Multivariate and stratified analyses, Kaplan–Meier method, and Cox proportional hazard regression were used to evaluate DM risk associated with the use of HCQ and glucocorticoid, both individually and in combination.

**Results:**

Among pSS patients (4,874 HCQ users and 2,437 HCQ nonusers), 497 patients developed DM over an average follow-up of 4.89 years. Multivariate analysis revealed significantly lower adjusted hazard ratios (aHRs) for DM in HCQ users in the 151–350 cumulative defined daily dose (cDDD) and ≥ 351 cDDD subgroups (0.600, 95% CI: 0.454–0.794 and 0.326, 95% CI: 0.246–0.433, respectively) compared with HCQ nonusers. High-dose glucocorticoids (≥ 151 cDDD) were linked to increased DM risk (aHR: 1.833, 95% CI: 1.410–2.383). However, high-dose HCQ (> 350 cDDD) mitigated this risk, even the risk caused by the use of high-dose glucocorticoids (≥ 151 cDDD) (aHR: 0.632, 95% CI: 0.421–0.948, *P* < 0.01).

**Conclusions:**

Our study demonstrated that HCQ exposure significantly reduces the risk of developing diabetes in patients with pSS. While higher doses of glucocorticoids are associated with an increased diabetes risk, concurrent HCQ use mitigates this risk in a dose-dependent manner.

## Introduction

Primary Sjögren syndrome (pSS) is an autoimmune disease characterized by lymphocytic infiltration of the exocrine glands, which leads to symptoms such as dry eyes and dry mouth [1, 2] . Despite ongoing research, the etiology of pSS is yet to be fully understood, complicating its management. The treatment regimen involves various pharmacological agents aimed at controlling symptoms and halting disease progression [3]. Hydroxychloroquine (HCQ), originally an antimalarial drug, has been widely used for treating autoimmune conditions such as systemic lupus erythematosus (SLE) and rheumatoid arthritis (RA). In pSS, HCQ is recommended for managing inflammatory musculoskeletal pain, fatigue, or as a steroid-sparing agent in the treatment of systemic active disease [3, 4]. The therapeutic potential of HCQ extends beyond symptom management; it has been found to decrease the risk of diabetes mellitus (DM) in patients with SLE or RA [5–8].

The effects of glucocorticoid use on glucose metabolism have been well-documented, and glucocorticoids significantly contribute to the development of DM.[7, 8] This poses a particular challenge in the management of pSS, because glucocorticoids are commonly prescribed to control inflammation and autoimmune activity in pSS. Adjunctive therapies that can mitigate the side effects of glucocorticoids on metabolism, including their impact on glucose homeostasis, should be identified [9–12].

Compared with research on RA and SLE, research focusing on SS, particularly pSS, is limited. SS is one of the autoimmune diseases commonly treated with glucocorticoids; glucocorticoid use may alter glucose metabolism and increase DM risk. The potential of HCQ to reduce diabetes risk in pSS and the influence of different dosages of the concurrent use of HCQ and glucocorticoids on diabetes risk have not been thoroughly investigated.

To address the aforementioned knowledge gap, we conducted a nationwide population-based study utilizing data from Taiwan’s National Health Insurance Research Database. This study assessed DM risk in pSS patients, focusing on the effects of HCQ use and the dose-dependent effects of concurrent HCQ and glucocorticoid use. The findings of this study can inform treatment decisions and promote the development of personalized therapeutic approaches for patients with pSS; thus, the study findings have significant implications for clinical practice. By gaining an understanding of the interactions between HCQ and glucocorticoids in the context of glucose metabolism, improved management strategies for pSS can be developed, reducing the incidence of glucocorticoid-induced DM.

## Methods

### Data source

Taiwan's National Health Insurance (NHI) program, which was launched by the Taiwanese government in March 1995, is a mandatory comprehensive insurance program covering > 96% of the population of Taiwan [13]. Enrollment is obligatory for all individuals registered in the census for more than 6 months, granting them access to outpatient, emergency room, and inpatient services. The National Health Research Institute manages the extensive electronic administrative datasets derived from the NHI program; these datasets are accessible to researchers after individual health data are de-identified. The protocol for this nationwide population-based retrospective cohort study received approval from the Ethics Institutional Review Board of Taipei Memorial Hospital (approval number: N201509007). This study adhered to the ethical standards outlined in the Declaration of Helsinki and was conducted in compliance with the Strengthening the Reporting of Observational Studies in Epidemiology (STROBE) guidelines.

### Study cohort

This retrospective cohort study included all patients in Taiwan diagnosed with Sjögren's syndrome (SS) between 2006 and 2015, identified using the International Classification of Diseases, 9 th Revision, Clinical Modification (ICD- 9-CM) code 710.2. In Taiwan, rheumatologists can apply for a catastrophic illness card for patients with SS who meet the diagnostic criteria established by the American–European Consensus Group (AECG). The application undergoes a thorough peer review process to ensure accuracy. Patients with SS who are approved for the catastrophic illness card are exempt from copayments. To identify SS patients in the claims data, we utilized the Registry for Catastrophic Illness Patients within the NHI database.The index date for each patient was defined as the date of their first ambulatory care visit with an SS diagnosis. Additionally, patients with prior diagnoses of RA (ICD- 9-CM code 714.0), SLE (ICD- 9-CM code 710.0), systemic sclerosis (ICD- 9-CM code 710.1), or polymyositis/dermatomyositis (ICD- 9-CM codes 710.3/710.4) were excluded. To ensure the inclusion of patients with a new diagnosis of SS, individuals with an index date before January 1, 2006, were excluded. Patients were also excluded if they had < 180 days of follow-up, had a diagnosis of DM (ICD- 9-CM code 250) before the index date, or had incomplete information. The final study cohort consisted of 9,348 pSS patients. The comorbid conditions recorded included hyperlipidemia (ICD- 9-CM code 272), hypertension (ICD- 9-CM codes 401–405), stroke (ICD- 9-CM codes 430–438), ischemic heart disease (ICD- 9-CM codes 410–414), and chronic kidney disease (ICD- 9-CM code 585). These comorbidities were documented if their diagnostic codes were present in two or more ambulatory claims within 12 months before the index date.

### Drug exposure

We collected detailed data on the drug type, dosage, administration route, prescription date, and total number of HCQ (ATC code: P01BA02) and glucocorticoids (ATC code: H02 AB) prescriptions fulfilled. Due to the possibility of drug use in separate years within the study period and potential changes in drug use patterns over time, we treated drug use as a time-varying covariate in our Cox proportional hazard model. The cumulative dose was calculated by multiplying the number of prescriptions fulfilled by the prescribed dose; this value was divided by the supply on the recorded days. Drug dosage was standardized as the defined daily dose (DDD). According to World Health Organization (WHO), DDD was defined as the average maintenance dose per day for a drug used for its main indication in adults. The DDD does not necessarily reflect the recommended or prescribed daily dose. The DDD recommended by the WHO was considered for HCQ (0.516 g/day for HCQ) and glucocorticoids (1.5 mg/day for betamethasone, 1.5 mg/day for dexamethasone, 10 mg/day for fluocortolone, 7.5 mg/day for methylprednisolone, 4 mg/day for paramethasone, 10 mg/day for prednisolone, 10 mg/day for prednisone, 7.5 mg/day for triamcinolone, 30 mg/day for hydrocortisone and 37.5 mg/day for cortisone).Patients with three or fewer prescriptions of the drugs were classified as nonusers, and those with more than three prescriptions of the drugs were classified as drug users. Additionally, for HCQ, we stratified patients into three subgroups by three cumulative DDD (cDDD) levels: 1 ≤ cDDD < 150, 150 ≤ cDDD < 350, and cDDD ≥ 350. For glucocorticoids, the three subgroups were defined by 1 ≤ cDDD < 40, 40 ≤ cDDD < 150, and cDDD ≥ 150.

### Study outcome

The primary outcome of this study was the incidence of DM. DM was defined based on the presence of at least two diagnostic codes for DM (ICD- 9-CM code 250) along with a new prescription for diabetes medication, including all insulin preparations and oral antidiabetic drugs, in the claims. Patients were monitored until the development of DM, permanent disenrollment from the NHI, or the end of the study on December 31, 2015, whichever occurred first.

### Statistical analysis

Elementary comparisons of categorical covariates were conducted through the chi-square test or Wilcoxon rank-sum test, and descriptive statistics were obtained for these covariates. Categorical covariates are expressed in terms of the proportion or median difference. Mean differences in continuous covariates were analyzed using the *t* test. To increase the power of statistical analysis, a propensity score–matching model was applied. The model included age, gender, and comorbid diabetes at baseline. Moreover, each patient without HCQ was propensity-score matched with two patients with HCQ at a 1:2 ratio. The hazard ratio (HR) of incident DM was calculated using univariate and multiple Cox proportional hazard models with 95% confidence interval (95% CI). Cumulative survival curves for DM were plotted using the Kaplan–Meier method, and statistical significance was determined using the log-rank test. Multivariate Cox proportional hazard models were used to identify independent factors contributing to the development of DM. All statistical analyses were performed using SAS version 9.4 (SAS Institute, Cary, NC, USA). All statistical tests were two-sided and were conducted at a significance level of 0.05. Moreover, P values and 95% CIs were reported for the tests.

## Results

### Study population characteristics

**Fig. 1:**
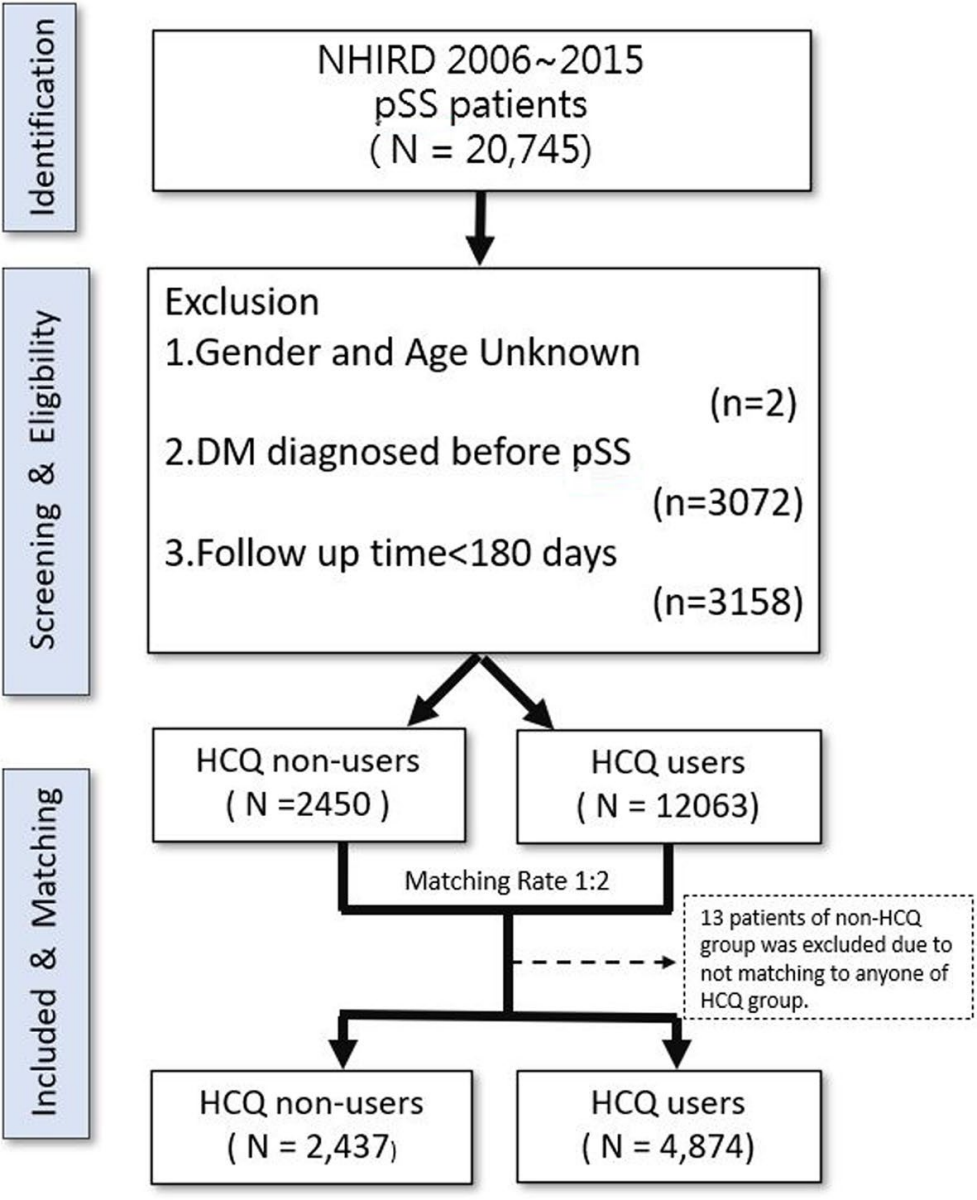
Flowchart of study design. NHIRD, National Health Insurance Research Database; pSS, primary Sjögren syndrome; HCQ, hydroxychloroquine; DM, diabetes mellitus

Figure 1 depicts the study flowchart. A total of 20,745 patients who received a diagnosis of pSS from 2006 to 2015 were included in this study. The exclusion criteria were as follows: (1) unknown gender or age, (2) a diagnosis of DM before pSS, and (3) a follow-up duration of < 180 days. Eligible individuals were divided into two groups: HCQ users (*n* = 12,063) and HCQ nonusers (*n* = 2,450). Each HCQ nonuser was propensity-score matched by age, gender, and comorbidities with two HCQ users at a 1:2 ratio. The final study sample comprised 2,437 HCQ nonusers and 4,874 HCQ users.

### Demographic characteristics of the pSS cohort

Table 1 outlines the demographic characteristics of the pSS cohort. The average follow-up period was 4.9 years (SD = 3.5 years). The cohort was predominantly female (85.3%), with a mean age of 54.9 years (SD = 14.8 years). Patients using HCQ had a longer follow-up duration than those not using HCQ [5.1 years (SD = 3.5 years) vs. 4.4 years (SD = 3.4 years), *P* < 0.001]. Additionally, HCQ users were administered higher doses of glucocorticoids than HCQ nonusers [256 cDDD (SD = 498) vs. 115 cDDD (SD = 363), *P* < 0.001]. The incidence rate of Type 2 diabetes was notably lower among HCQ users than among HCQ nonusers (5.54% vs. 6.69%, *P* = 0.049) and the incidence of overall diabetes was also significantly lower among HCQ user than among HCQ nonuser (6.23% vs. 7.51%, *P* = 0.039).

**Table 1:**
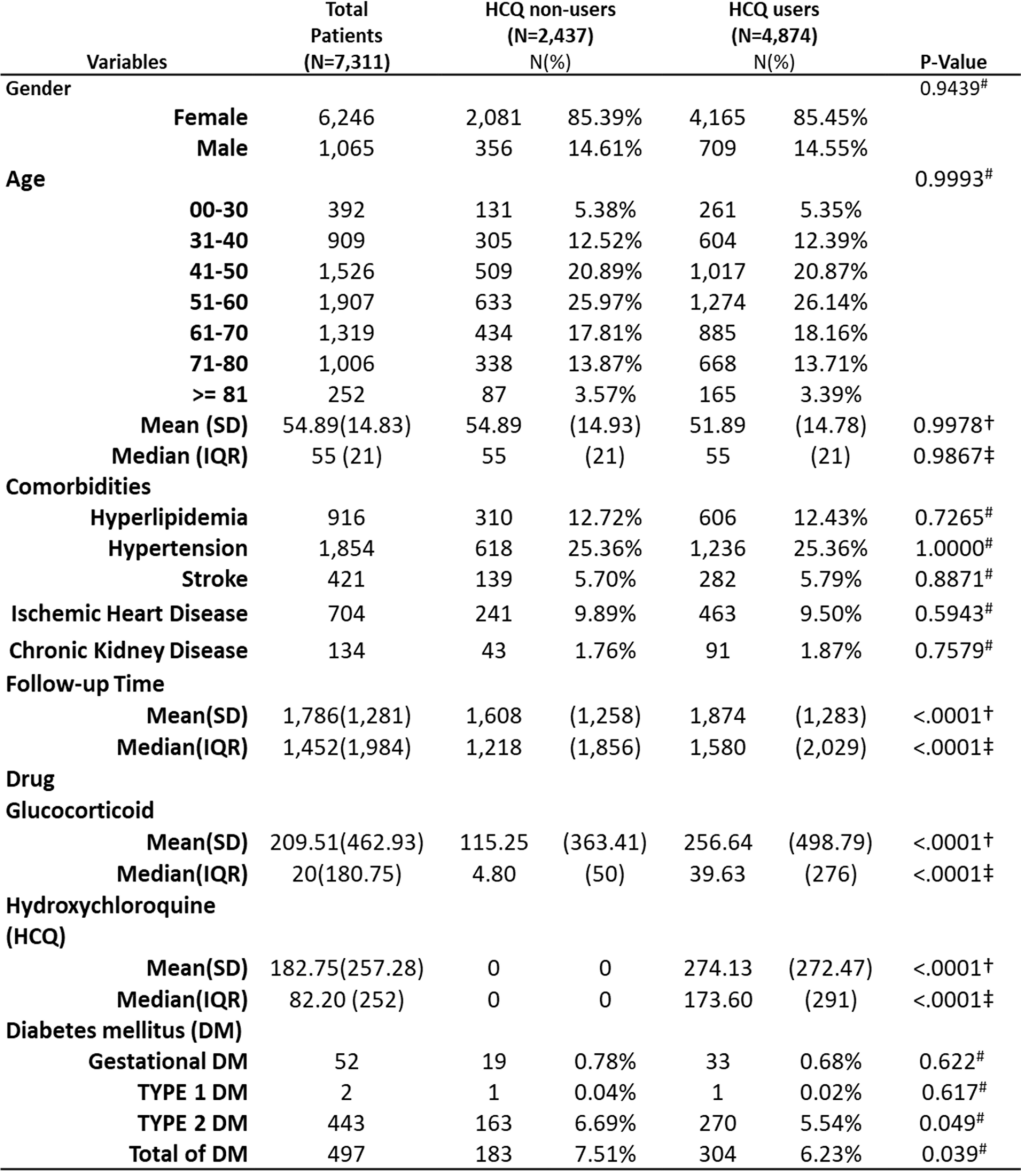
Baseline characteristics of pSS patients with and without HCQ use

# P-value was estimated by Chi-square test, + P-value was estimated by t test, ± P-value was estimated by Kruskal-Wallis test, pSS: primary Sjögren syndrome

### Effect of HCQ/glucocorticoids on incident DM

**Fig. 2:**
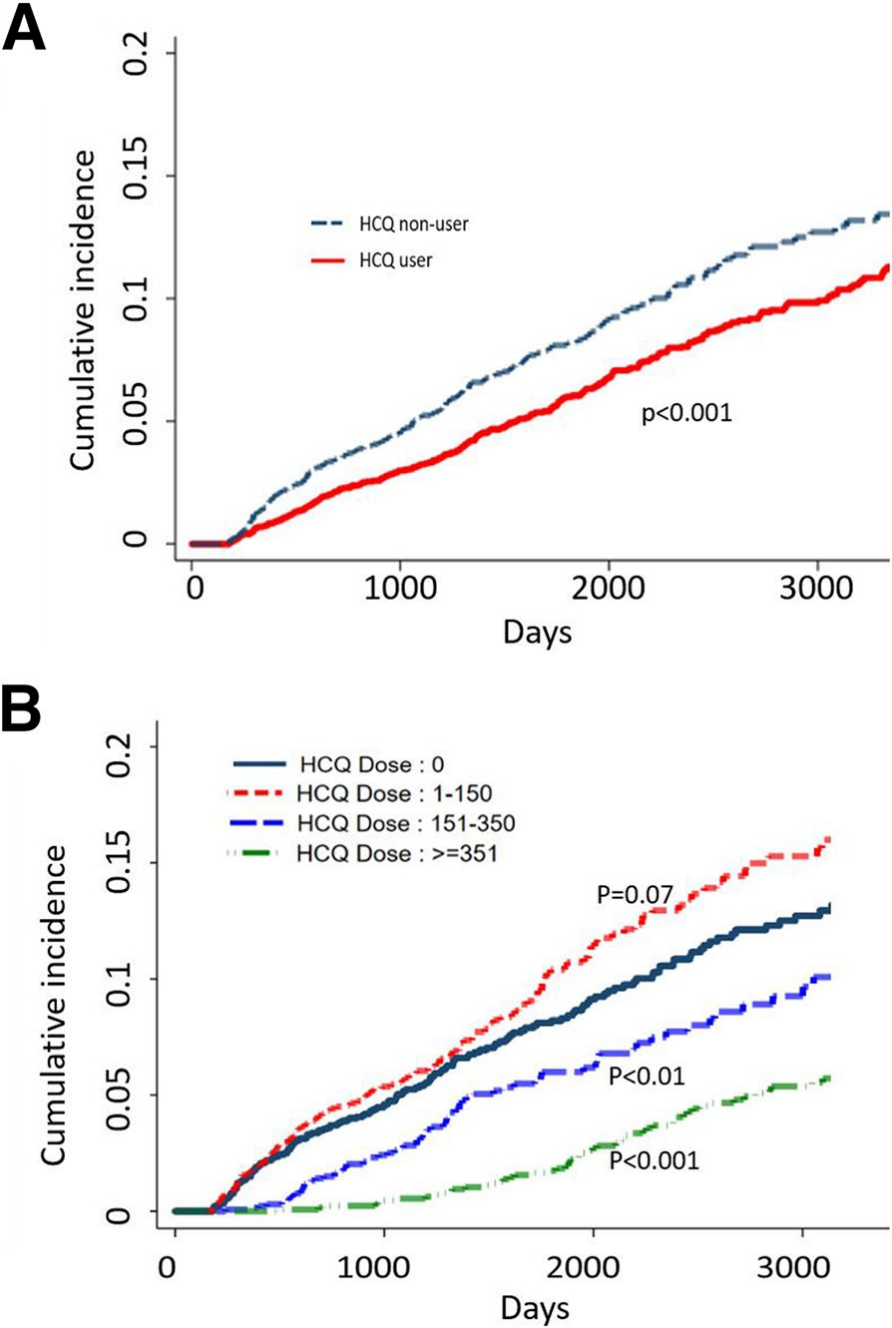
**A** Cumulative risk analysis of HCQ users and HCQ nonusers. **B** Cumulative risk analysis of different dosages of HCQ, HCQ, hydroxychloroquine

To study the effects of the drugs of interest on the incidence of DM in pSS patients, we calculated the HRs for diabetes development (Table 2). In the multivariable analysis, no significant difference was found in diabetes incidence by gender, whereas older age tended to be associated with increased diabetes incidence (without statistical significance). Regarding the effects of HCQ, compared with patients who did not use HCQ, those using HCQ at the doses of 151–350 cDDD and ≥ 351 cDDD had a significantly reduced risk of diabetes. The adjusted HRs (aHRs) were 0.600 (95% CI: 0.454–0.794) and 0.326 (95% CI: 0.246–0.433), respectively (Table 2). The Kaplan–Meier survival analysis revealed a lower cumulative risk of diabetes in HCQ users than in nonusers, with an adjusted HR (aHR) of 0.727 (95% CI: 0.607–0.871) (Fig. 2A). Figure 2B illustrates the differences in the reduction of diabetes risk across different doses of HCQ. The results indicated that higher doses of HCQ (≥ 151 cDDD) were associated with a lower risk of diabetes. Additionally, the use of glucocorticoids led to increases in the incidence of diabetes. A higher risk of diabetes was associated with higher glucocorticoid doses. Compared with patients who did not use glucocorticoids, those who used glucocorticoids at the doses of ≥ 151 cDDD exhibited statistically significantly increased diabetes risk (aHR: 1.833, 95% CI: 1.410–2.383) (Table 2).

**Table 2:** Association of HCQ, concurrent medications, and comorbidities with DM risk

	**HCQ nonusers**		**HCQ users**		**Univariate Analysis**		**Multivariate Analysis**	
	N	Events of DM	N	Events of DM	HR	95% C.I	ADJ. HR	95% C.I
**Overall**	2,437	183	4,874	314	1.000			
					0.727**	(0.607–0.871)		
**HCQ (cDDD)**								
**No Use (ref.)**	2,437	183	0	0	1.000		1.000	
**1–150**	0	0	2,197	164	1.217	(0.985–1.505)	1.161	(0.937–1.439)
**151–350**	0	0	1,367	76	0.660**	(0.504–0.865)	0.600**	(0.454–0.794)
≥ **351**	0	0	1,310	74	0.403***	(0.309–0.524)	0.326***	(0.246–0.433)
**Glucocorticoid (cDDD)**								
**No Use (ref.)**	1,056	44	1,347	59	1.000		1.000	
**1–40**	692	63	1,092	60	1.109	(0.851–1.444)	1.145	(0.877–1.496)
**41–150**	322	26	803	53	1.146	(0.850–1.546)	1.161	(0.856–1.574)
≥ **151**	367	50	1,632	142	1.344*	(1.053–1.715)	1.833***	(1.410–2.383)
**Gender**								
**Female (ref.)**	2,081	153	4,165	263	1.000		1.000	
**Male**	356	30	709	51	1.153	(0.899–1.478)	0.879	(0.683–1.132)
**Age group**								
**00–30 (ref.)**	131	4	261	19	1.000		1.000	
**31–40**	305	22	604	19	0.770	(0.449–1.320)	0.798	(0.465–1.370)
**41–50**	509	38	1017	48	0.999	(0.617–1.618)	1.010	(0.621–1.641)
**51–60**	633	43	1274	74	1.229	(0.767–1.969)	1.137	(0.705–1.833)
**61–70**	434	43	885	76	1.835*	(1.142–2.950)	1.566	(0.960–2.556)
**71–80**	338	25	668	65	1.885**	(1.167–3.047)	1.336	(0.805–2.216)
> ** = 81**	87	8	165	13	2.470***	(1.338–4.559)	1.558	(0.825–2.940)
**Comorbidities**								
**Hyperlipidemia**	310	31	606	55	1.631***	(1.285–2.071)	1.241	(0.966–1.594)
**Hypertension**	618	66	1236	118	1.955***	(1.628–2.348)	1.394**	(1.109–1.752)
**Stroke**	139	21	282	31	2.092***	(1.579–2.772)	1.476*	(1.084–2.009)
**Ischemic Heart Disease**	241	27	463	50	1.812***	(1.427–2.300)	1.193	(0.919–1.549)
**Chronic Kidney Disease**	43	6	91	12	2.763***	(1.879–4.063)	1.844**	(1.244–2.735)

*HCQ* Hydroxychloroquine, *cDDD* cumulative defined daily dose

^*^ 0.01 ≤ *P* < 0.05, ** 0.0001 ≤ *P* < 0.01, *** *P* < 0.0001

### Analysis of interaction risk between HCQ and glucocorticoids

Table 3 presents the results of Cox proportional hazard analysis of the interactions between varying doses of HCQ and glucocorticoids in relation to diabetes risk. The analysis revealed that in the absence of HCQ use, high doses of glucocorticoids (≥ 151 cDDD) resulted in a significantly increased risk of DM (aHR: 2.058, 95% CI: 1.365–3.102). When patients were prescribed HCQ doses ranging from 0 to 350 cDDD in conjunction with high doses of glucocorticoids, the risk of diabetes remained elevated (aHR: 1.790, 95% CI: 1.247–2.568). Notably, the aHRs for DM risk in patients without, with low or high glucocorticoid doses in conjunction with HCQ > 350 cDDD were 0.442 (95% CI: 0.201–0.973), 0.487 (95% CI: 0.294–0.806), and 0.632 (95% CI: 0.421–0.948), respectively. Even when administered high doses of glucocorticoids (≥ 151 cDDD), patients exhibited a significantly reduced risk of DM when also using HCQ at the doses of > 350 cDDD.

**Table 3:**
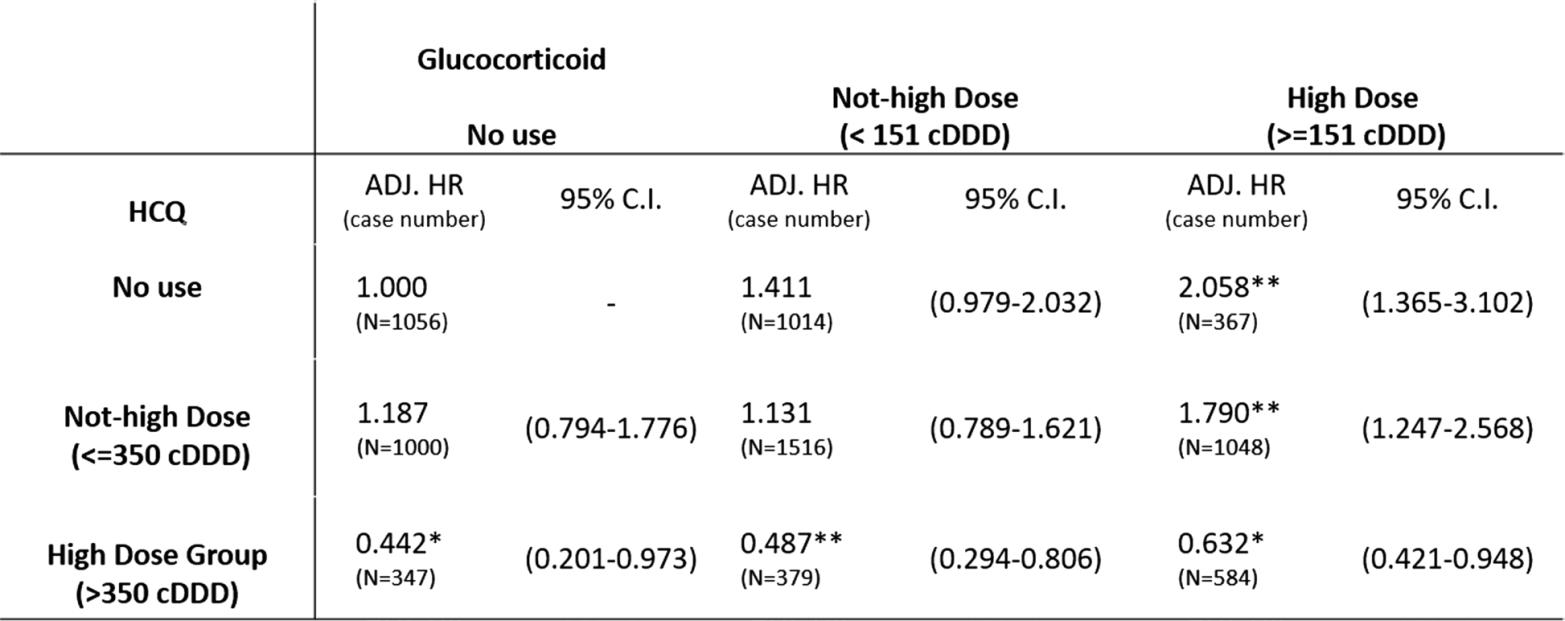
Interaction hazard risk analysis betwee HCQ and steroid dose group

All model were adjusted by disease, gender, age group and comorbidities.

*HCQ* Hydroxychloroquine, *cDDD*  cumulative defined daily dose

### Association of age, gender, and comorbidities with diabetes risk in pSS patients

Table 4 presents the results of a subgroup analysis of risk factors for diabetes according to age, gender, and comorbidities. Female patients had a lower risk of diabetes, with an aHR of 0.667 (95% CI: 0.551–0.833). In terms of age, the 41–55 years and 56–70 years groups exhibited lower risks of diabetes, with aHRs of 0.550 (95% CI: 0.391–0.774) and 0.694 (95% CI: 0.507–0.948), respectively. Among comorbidities, only stroke was associated with a significantly lower risk of diabetes, whereas no statistical differences were observed for other comorbidities.

**Table 4:**
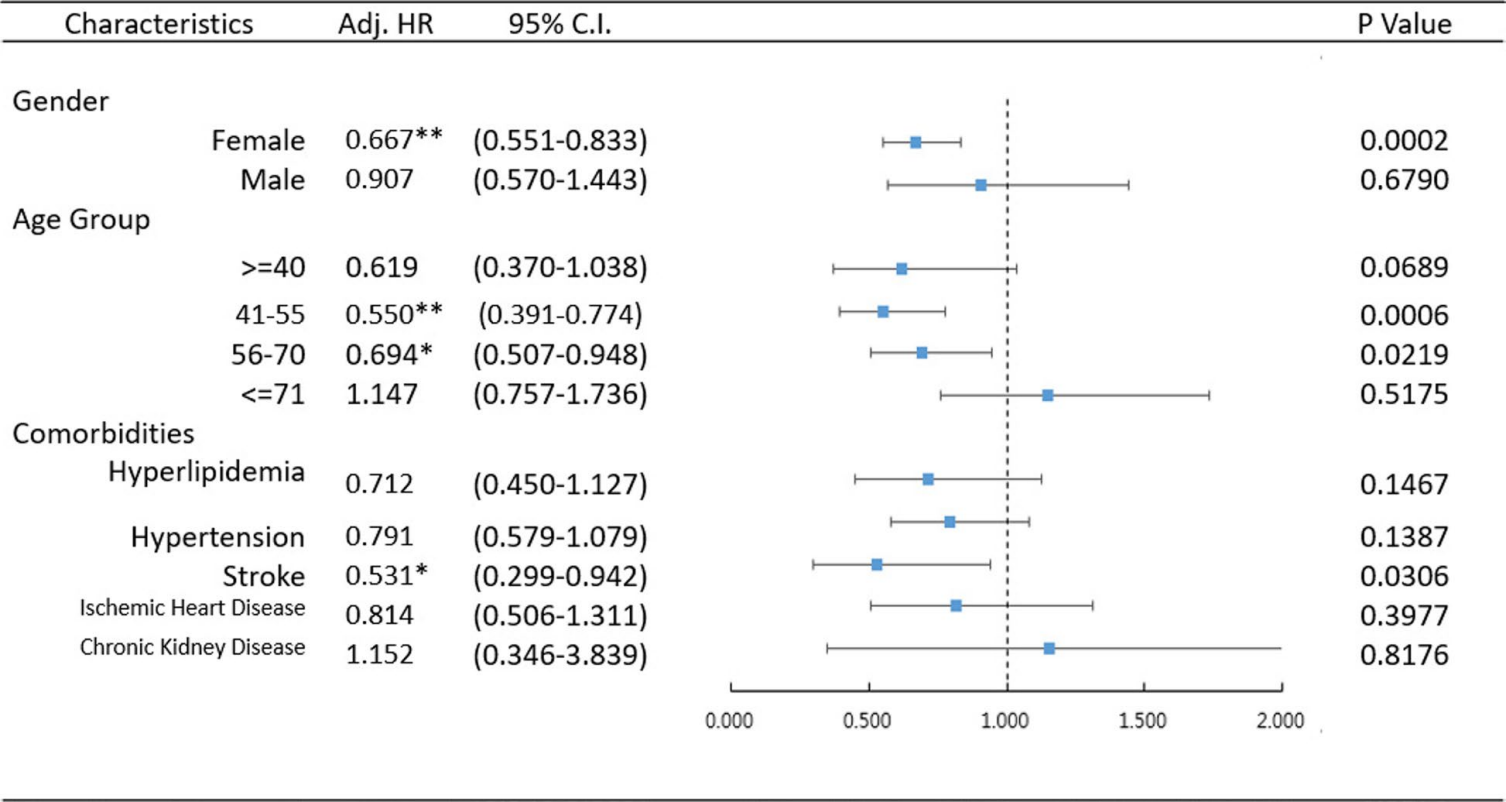
All model were adjusted by gender, age group and comorbidities

* 0.01 <= *P* Value <0.05, ** 0.0001<= *P* Value <0.01

## Discussion

This is the first population-based cohort study to demonstrate that HCQ use dose-dependently reduces the risk of diabetes among pSS patients caused by high-dose glucocorticoids use. The use of glucocorticoids at doses exceeding 151 cDDD led to an increased risk of diabetes. However, the concurrent use of HCQ at doses of > 350 cDDD could mitigate the potential adverse effects of high-dose glucocorticoids on diabetes risk.

Increasing evidence suggests that HCQ can regulate glucose homeostasis, regardless of the presence or absence of diabetes [14, 15]. However, the mechanisms through which HCQ decreases blood glucose levels remain unclear. Currently, HCQ is believed to influence glucose homeostasis through various mechanisms, including improving insulin sensitivity, increasing insulin secretion, and reducing systemic inflammation [14, 16, 17]. Given its glucose-lowering properties, some studies have found that the adjuvant use of HCQ can significantly help control blood glucose in T2DM patients who are already using two to three antidiabetic medications that did not provide adequate control [17–19]. Additionally, Wasko et al. found that HCQ at a dose of 400 mg/day can increase the plasma levels of adiponectin [16]; through its anti-inflammatory effects, adiponectin plays a key role in insulin resistance and metabolic syndrome development by enhancing insulin sensitivity [20–22]. Chronic low-grade inflammation and islet inflammation are associated with insulin resistance and β-cell dysfunction in type 2 diabetes, respectively. HCQ may alleviate inflammation by controlling toll-like receptors, reducing oxyradical release, and decreasing cytokine production. The anti-inflammatory effects of HCQ may bridge its insulin-sensitizing and insulinotropic properties [23].

Consistent with our results, previous studies have also reported that HCQ reduces the incidence of diabetes in other inflammatory rheumatic diseases [7, 8]. Additionally, clinical research supports the use of HCQ for improving glucose metabolism in patients with rheumatic diseases, and HCQ also has beneficial effects in patients with cancer, infections, and dyslipidemia [24, 25]. However, a recent study demonstrated that the overall prevalence of HCQ retinopathy was 7.5% but varied with daily consumption (> 5.0 mg/kg) and with duration of use (> 10 years) [26, 27]. Thus, clinicians should be aware of these side effects when administering higher daily dosage and longer use of HCQ [28].

In recent years, cardiovascular disease has become a leading cause of mortality in many autoimmune diseases. YJ Su et al. demonstrated that, in addition to hypertension and hyperlipidemia, SS is an independent risk factor for cardiovascular events in diabetic patients (adjusted odds ratio [aOR]: 1.67, 95% CI: 1.06–2.65) [29]. In contrast, other autoimmune diseases such as RA, ankylosing spondylitis(AS), and SLE were not identified as significant risk factors in the same study.This suggests that reducing diabetes risk through the use of HCQ may hold greater clinical significance in pSS than in other autoimmune diseases. Our findings indicate that, even when combined with glucocorticoids, an HCQ cumulative dose of ≥ 350 cDDD effectively reduces the incidence of diabetes. From the perspective of cardiovascular risk, this cumulative dosage underscores the critical role of HCQ in the management of pSS.

Glucocorticoids exacerbate hyperglycemia; various small and large studies have reported that the prevalence of hyperglycemia ranges from 5 to 45% in patients using glucocorticoids (OR: 1.3–2.7) [30]. According to the EULAR treatment guidelines for SS, the dosage and duration of corticosteroids should be minimized, and immunosuppressive agents should primarily be used as steroid-sparing agents [3]. This study confirms that HCQ can reduce the risk of steroid-induced diabetes. Thus, the results serve a significant reference for rheumatologists for selecting csDMARDs for pSS patients who require long-term glucocorticoid therapy.

Our study has several strengths. First, compared with previous studies on SS, rather than simply classifying patients as users and nonusers, this study adjusted for the dosages of HCQ. Second, our sample size is the largest across all studies examining the effect of HCQ on diabetes in pSS patients. Third, a previous study investigated secondary SS, which often coexists with autoimmune diseases such as RA and SLE [31]. Varying risks of diabetes have been reported for these autoimmune diseases, and the diseases involve more complex pathological mechanisms and treatments that may affect study outcomes. By focusing solely on pSS and excluding secondary SS, the risk of unnecessary bias was reduced in our study. In addition, a drug prescription does not necessarily indicate that the patient took the medication or adhered to the prescribed dose and duration. We attempted to mitigate this problem by conducting analyses based on the cDDD. The results of this study demonstrated that with the increasing cDDD of HCQ, the risk of diabetes decreased, indicating the robustness of our data. Lastly, previous SS studies have not considered the effects of glucocorticoids, a significant cause of diabetes. However, our study explored the interaction between HCQ and corticosteroids in relation to diabetes risk.

This study has several limitations. First, this study was conducted using data from the Taiwan’s NHI Research Database. Although this database contains the claims data of a considerable portion of the population of Taiwan, it lacks basic information, such as patients'laboratory data, which imposes restrictions on the accuracy and completeness of data. Information on the disease activity of pSS and the laboratory data for HCQ users and nonusers are unavailable, limiting our exploration of the relationship between pSS disease activity and the risk of diabetes. Second, information on lifestyle factors, BMI, and family medical history is lacking in the database, which may influence the risk of diabetes. Thirdly, the incidence of diabetes varies across populations with different ethnic backgrounds [32]. Since the study population in this database primarily consists of individuals of Asian ethnicity, the findings of this study may not be generalizable to pSS patients from diverse ethnic backgrounds. Lastly, although this study provides insights into the potential protective effects of HCQ on diabetes risk in pSS patients, a definitive causal relationship could not be established in this study. The results underscore the need for further research, including prospective studies and clinical trials, to corroborate these findings and to explore the underlying mechanisms.

## Conclusions

We conducted a nationwide survey to examine the association between HCQ use and DM risk in pSS patients, focusing on the dose-dependent effects of concurrent HCQ and glucocorticoid use on the development of diabetes in these patients. The results indicated that pSS patients using high doses of HCQ had a significantly low risk of diabetes, whereas the use of high doses of glucocorticoids significantly increased the risk of diabetes. However, the concomitant use of high doses of HCQ could mitigate the diabetes risk induced by the use of high doses of glucocorticoids. Further research is needed to explore the mechanisms underlying the beneficial effects of HCQ and its long-term effects.

## Data Availability

The datasets used or analyzed during the present study are available from the corresponding author upon reasonable request.
